# A Continuous Severity Index for Keratoconus Diagnosis Based on Kolmogorov–Arnold Networks in a Chinese Population

**DOI:** 10.1016/j.xops.2026.101168

**Published:** 2026-03-23

**Authors:** Ying Qiao, Lin Wang, Lie Ju, Ming Hu, Jingbin Geng, Zishuo Li, Ling Sun, Lan Ding, Mi Tian, Chao Hu, Zongyuan Ge, Xiaoying Wang, Xingtao Zhou, Yang Shen, Xiaoyu Zhang

**Affiliations:** 1 School of Health Science and Engineeering, University of Shanghai for Science and Technology, Shanghai, China; 2 Department of Ophthalmology, EYE & ENT Hospital of Fudan University, Shanghai, China; 3 Key Laboratory of Myopia and Related Eye Diseases, NHC, Shanghai, China; 4 Key Laboratory of Myopia and Related Eye Diseases, Chinese Academy of Medical Sciences, Shanghai, China; 5 Institute of Ophthalmology, University College London, London, UK; 6 NIHR Biomedical Research Centre at Moorfields Eye Hospital NHS Foundation Trust, London, UK; 7 Faculty of Engineering, Department of Electrical and Computer Systems, Monash University, Melbourne, Australia; 8 China Unicom (Shanghai) Industry Internet Company Ltd., Shanghai, China

**Keywords:** Keratoconus, Forme fruste keratoconus, Corneal biomechanics, cCBI, Kolmogorov–Arnold Network

## Abstract

**Objective:**

To develop and validate a continuous, interpretable severity index for keratoconus (KC) using a Kolmogorov–Arnold Network (KAN) to improve early detection and disease staging, particularly for forme fruste keratoconus (FFKC) in refractive surgery screening.

**Design:**

A retrospective case-control study.

**Participants:**

A total of 384 eyes from 384 participants were included: 101 keratoconic eyes, 132 FFKC eyes (fellow eyes of KC patients), and 151 normal control eyes derived from refractive surgery candidates with at least 2 years of uneventful follow-up.

**Methods:**

Corneal tomographic parameters were obtained using Pentacam HR, and biomechanical parameters using Corvis ST. A KAN model was trained using categorical diagnostic labels (normal, FFKC, and KC) to generate a continuous, dimensionless Continuous Severity Index (CSI). Continuous Severity Index performance was compared with established indices, including Corvis Biomechanical Index, Corvis Biomechanical Index for Chinese populations, Tomography and Biomechanical Index, Tomography and Biomechanical Index for Chinese populations, and Stress–Strain Index, using receiver operating characteristic analysis. Feature attribution analysis was performed to explore stage-dependent parameter contributions.

**Main Outcome Measures:**

Area under the receiver operating characteristic curve (AUC), sensitivity, specificity, and optimal cutoff values for differentiating disease stages.

**Results:**

Continuous Severity Index achieved an AUC of 1.000 for distinguishing KC from normal eyes, and 0.859 for distinguishing FFKC from normal eyes, outperforming all conventional biomechanical and tomographic indices. In combined screening tasks, CSI demonstrated superior performance in identifying any ectatic change (FFKC + KC vs. normal, AUC = 0.920) and advanced disease (KC vs. normal + FFKC, AUC = 0.998). Feature attribution analysis further revealed a stage-dependent shift in dominant contributors, with biomechanical parameters prevailing at lower CSI levels and tomographic asymmetry features increasingly governing advanced disease.

**Conclusions:**

The KAN-derived Continuous Severity Index enables unified, continuous, and interpretable quantification of KC severity, outperforming existing indices in both early screening and disease staging. By capturing the biologically coherent progression from biomechanical instability to morphological deformation, CSI provides a clinically meaningful tool for refractive surgery screening and KC management.

**Financial Disclosure(s):**

The authors have no proprietary or commercial interest in any materials discussed in this article.

Keratoconus (KC) is a progressive corneal ectatic disorder characterized by localized thinning and anterior protrusion of the cornea, leading to irregular astigmatism and visual impairment. Its global prevalence is estimated to range from 0.1% to 2%, highlighting its significance as a common cause of visual morbidity worldwide.^1^^,^^2^ Early detection of KC is of paramount importance, particularly in the context of corneal refractive surgery, as undiagnosed early-stage disease remains a major risk factor for postoperative ectasia.^3^

However, diagnosing early or subclinical KC—commonly referred to as forme fruste keratoconus (FFKC)—remains a substantial clinical challenge. In this stage, classical tomographic features are often absent or subtle, rendering traditional morphology-based screening tools insufficient. Increasing evidence suggests that biomechanical weakening precedes detectable morphological changes, highlighting the diagnostic value of corneal biomechanical assessment.^4^

The Corvis ST (OCULUS) Biomechanical Index (CBI) and the Tomography and Biomechanical Index (TBI) represent important advances in integrating biomechanical and tomographic data.^5^^,^^6^ Nevertheless, these indices rely on predefined thresholds and discrete classification paradigms, which inherently limit their ability to model the continuous and progressive nature of KC. Furthermore, population-specific biomechanical differences—particularly in Asian cohorts—necessitate localized recalibration,^7^, ^8^, ^9^ as reflected by the development of cCBI and cTBI for Chinese populations.^10^

Beyond population differences, a more fundamental limitation persists: KC is a continuous disease spectrum, yet current diagnostic tools primarily operate in a categorical framework. This mismatch may partially explain the persistent diagnostic uncertainty surrounding FFKC.

Kolmogorov–Arnold Networks (KANs)^11^ represent a recently introduced class of neural networks that replace fixed linear weights with learnable univariate spline functions, offering enhanced representational flexibility and interpretability. Unlike conventional multilayer perceptron or ensemble tree methods, KANs are particularly well suited for modeling structured biomedical data with complex but smooth nonlinear relationships.

In this study, we hypothesize that a KAN-based model can effectively integrate corneal tomographic and biomechanical parameters to construct a continuous severity index for KC. We aim to (1) develop a KAN-derived Continuous Severity Index (CSI), (2) evaluate its diagnostic performance across the full disease spectrum, and (3) compare its performance with established indices in a Chinese population.

## Methods

### Study Design and Ethics

This single-center retrospective case-control study was approved by the Ethics Committee of the Eye & ENT Hospital of Fudan University (Approval No. [2023108-1]) and adhered to the tenets of the Declaration of Helsinki. Written informed consent was obtained from all participants.

In the literature, the terms FFKC and subclinical KC partially overlap in definition. In this study, FFKC was defined as the clinically unaffected fellow eye of a patient with manifest KC, characterized by the absence of slitlamp signs of KC but potentially subtle tomographic abnormalities.

A total of 384 eyes were retrospectively enrolled, including 101 keratoconic eyes (KC), 132 FFKC eyes, and 151 normal eyes (NORMAL). All subjects underwent Corvis ST (Oculus Optikgeräte GmbH) and Pentacam HR (Oculus Optikgeräte GmbH) examinations at the Eye & ENT Hospital of Fudan University between December 2014 and February 2025, with complete clinical records and follow-up data.

For each subject, only 1 eye was included to avoid intereye correlation: 1 randomly selected eye from KC patients, the fellow eye from KC patients for the FFKC group, and 1 randomly selected eye from refractive surgery candidates with at least 2 years of uneventful follow-up for the NORMAL group.

Conventional Scheimpflug-derived indices (e.g., CBI and TBI) rely on manually engineered geometric and thickness thresholds and are essentially linear or ensemble-based decision boundaries. In contrast, this study introduces a KAN to jointly model tomographic and biomechanical parameters, replacing fixed linear weights with learnable spline-based functional mappings, thereby enabling a more flexible and interpretable continuous severity representation.

### Inclusion and Exclusion Criteria

#### Inclusion Criteria

KC group: Diagnosis of KC in both eyes, with characteristic tomographic features (inferior steepening or asymmetric bow-tie pattern) and ≥1 slitlamp sign (Munson sign, Vogt striae, Fleischer ring, corneal thinning, or Rizzuti sign).^12^

FFKC group: Fellow eye of a confirmed KC patient, presenting normal slitlamp findings and no diagnostic tomographic criteria for KC.^13^

NORMAL group: Normal findings on slitlamp examination and corneal tomography, derived from refractive surgery candidates with ≥2 years of postoperative follow-up without ectasia.^14^

#### Exclusion Criteria

Eyes were excluded if subjects had:

1. Contact lens wear within 4 weeks (rigid) or 2 weeks (soft),
2. Pregnancy or lactation,
3. Previous corneal refractive or transplant surgery,
4. Systemic or ocular diseases affecting corneal structure.

### Ophthalmic Examinations

All participants underwent comprehensive ophthalmic evaluation, including uncorrected visual acuity, best spectacle-corrected distance visual acuity, manifest and cycloplegic refraction, slitlamp biomicroscopy, corneal tomography (Pentacam HR), and corneal biomechanics (Corvis ST). Only measurements with acceptable quality scores were included in the analysis.^13^

### KAN-Based CSI

The proposed KAN model was trained using only categorical supervision (NORMAL, FFKC, and KC), without explicit severity labels. Input features included demographic, biomechanical, and tomographic parameters:

Demographic parameter: Age.

Biomechanical parameters: Ambrósio relational thickness (ARTh), stiffness parameter at first applanation (SP-A1).

Tomographic parameters: flat keratometry (K1 F), steep keratometry (K2 F), Pachy Min, Index of Surface Variance (ISV), Index of Vertical Asymmetry (IVA), Keratoconus Index (KI), Central Keratoconus Index (CKI), Index of Horizontal Asymmetry (IHA), Index of Height Decentration (IHD).

The KAN outputs a continuous, dimensionless severity score (CSI), embedding discrete disease states into a unified latent severity space. The resulting CSI was benchmarked against established biomechanical and tomographic indices to evaluate diagnostic performance across different disease stages.

1. Data preprocessing pipeline:
  - Missing values were imputed using the median of the training fold,
  - Features were standardized to zero mean and unit variance,
  - All preprocessing steps were confined within each cross-validation fold to prevent data leakage.
2. Severity index definition

Categorical labels were mapped to an ordinal scale and anchored at Normal = 0.0, FFKC = 0.5, and KC = 1.0. The model output class probabilities, and the severity score was defined as the expected ordinal level on the continuous interval [0, 1]:

S=0×P_Normal+0.5×P_FFKC+1.0×P_KC

This formulation preserves ordinal structure while enabling continuous assessment of disease severity.

3. Modeling and training

The hidden dimension of KAN is set to 25. To enforce ordinal consistency, we adopted a cumulative ordinal learning framework, ensuring that P(y > KC) ≤ P(y > FFKC). This approach improves class separation without directly regressing on the crafted severity target.

The KAN was trained with multiple synergistic objectives.

- Ordinal classification on cumulative thresholds with focal loss;
- Anchor loss, aligning each class toward its predefined severity anchor (0, 0.5, 1);
- Hinge gap loss, enforcing a minimum separation between adjacent class means.

### Statistical Analysis

Statistical analyses were performed using SPSS version 25.0. Continuous variables are presented as mean ± standard deviation.

1. Group comparisons among NORMAL, FFKC, and KC were conducted using 1-way analysis of variance, followed by Tukey honestly significant difference post hoc tests.
2. Diagnostic performance was evaluated using receiver operating characteristic analysis, with area under the receiver operating characteristic curve (AUC), sensitivity, specificity, and optimal cutoff values determined by the Youden index.

A 2-sided *P* value < 0.05 was considered statistically significant.

## Results

### Baseline Characteristics

Baseline demographic, tomographic, and biomechanical parameters are summarized in Table 1. A clear monotonic trend from NORMAL → FFKC → KC was observed for most biomechanical indices. Notably, Pentacam-derived irregularity indices (ISV, IVA, KI, CKI, IHA, IHD, K1 F, and K2 F) showed no statistically significant differences between FFKC and NORMAL eyes (*P* > 0.05), whereas biomechanical parameters (ARTh, SP-A1, cCBI, and cTBI) demonstrated significant separation, underscoring the early sensitivity of biomechanical alterations.

**Table 1:** Baseline Demographic, Tomographic, and Biomechanical Characteristics of Eyes with Keratoconus (KC), Forme Fruste Keratoconus (FFKC), and Normal Controls

	KC (n = 101)	FFKC (n = 132)	Normal (n = 151)	Overall	KC vs. Normal	FFKC vs. Normal	KC vs. FFKC
Age	29.80 ± 6.26 (15.00, 51.00)	28.80 ± 6.65 (16.00, 54.00)	32.56 ± 5.92 (23.00, 48.00)	<0.01	<0.01 (0.002)	<0.01	0.45
ARTh	211.48 ± 99.47 (49.83, 504.25)	424.76 ± 125.60 (62.44, 889.03)	509.16 ± 97.06 (344.22, 910.70)	<0.01	<0.01	<0.01	<0.01
SP A1	69.33 ± 22.85 (10.24, 165.74)	97.15 ± 18.42 (60.26, 154.21)	112.95 ± 15.94 (75.08, 177.37)	<0.01	<0.01	<0.01	<0.01
CBI	0.94 ± 0.14 (0.12, 1.00)	0.64 ± 0.28 (0.01, 1.00)	0.37 ± 0.21 (0.00, 0.76)	<0.01	<0.01	<0.01	<0.01
cCBI	0.93 ± 0.20 (0.02, 1.00)	0.46 ± 0.37 (0.00, 1.00)	0.17 ± 0.19 (0.00, 0.79)	<0.01	<0.01	<0.01	<0.01
TBI	0.98 ± 0.08 (0.44, 1.00)	0.70 ± 0.30 (0.02, 1.00)	0.31 ± 0.17 (0.01, 0.73)	<0.01	<0.01	<0.01	<0.01
cTBI	0.96 ± 0.16 (0.05, 1.00)	0.48 ± 0.35 (0.00, 1.00)	0.06 ± 0.04 (0.00, 0.24)	<0.01	<0.01	<0.01	<0.01
SSI	0.68 ± 0.22 (0.32, 2.04)	0.86 ± 0.16 (0.42, 1.36)	0.96 ± 0.13 (0.43, 1.31)	<0.01	<0.01	<0.01	<0.01
K1 F (D)	49.17 ± 6.82 (39.70, 72.20)	42.64 ± 1.56 (38.00, 46.80)	42.64 ± 1.39 (37.70, 47.00)	<0.01	<0.01	1	<0.01
K2 F (D)	52.99 ± 7.68 (40.10, 77.90)	43.91 ± 1.73 (39.10, 48.10)	43.97 ± 1.42 (38.90, 47.90)	<0.01	<0.01	0.991	<0.01
Pachy Min	445.92 ± 53.52 (277.00, 538.00)	513.01 ± 32.40 (423.00, 593.00)	541.79 ± 28.41 (487.00, 624.00)	<0.01	<0.01	<0.01	<0.01
ISV	97.85 ± 46.87 (29.00, 228.00)	21.10 ± 7.42 (9.00, 58.00)	18.12 ± 4.50 (7.00, 38.00)	<0.01	<0.01	0.566	<0.01
IVA	0.84 ± 0.45 (0.10, 2.70)	0.17 ± 0.09 (0.05, 0.57)	0.12 ± 0.04 (0.04, 0.23)	<0.01	<0.01	0.149	<0.01
KI	1.26 ± 0.16 (1.05, 1.89)	1.03 ± 0.03 (0.95, 1.14)	1.03 ± 0.02 (0.97, 1.09)	<0.01	<0.01	0.960	<0.01
CKI	1.10 ± 0.08 (0.97, 1.33)	1.01 ± 0.01 (0.98, 1.04)	1.01 ± 0.01 (1.00, 1.02)	<0.01	<0.01	0.872	<0.01
IHA	30.64 ± 24.82 (0.50, 109.80)	7.32 ± 5.73 (0.00, 26.00)	5.66 ± 4.05 (0.00, 20.80)	<0.01	<0.01	0.551	<0.01
IHD	0.13 ± 0.08 (0.02, 0.37)	0.02 ± 0.01 (0.00, 0.07)	0.01 ± 0.00 (0.00, 0.03)	<0.01	<0.01	0.439	<0.01

ARTh = Ambrósio relational thickness, reflecting the rate of corneal thickness increase from the thinnest point toward the nasal–temporal periphery, with lower values indicating thinner corneas or steeper peripheral thickening; CBI = Corvis Biomechanical Index; cCBI = Corvis Biomechanical Index for Chinese populations; CKI = Central Keratoconus Index, emphasizing central corneal curvature abnormalities and increased sensitivity to early central keratoconus; cTBI = Tomography and Biomechanical Index for Chinese populations; IHA = Index of Horizontal Asymmetry, measuring deviation from horizontal symmetry; IHD = Index of Height Decentration, describing decentration of the point of maximum corneal elevation; ISV = Index of Surface Variance, quantifying overall irregularity of corneal curvature; IVA = Index of Vertical Asymmetry, measuring deviation from vertical symmetry and capturing typical inferior–superior asymmetry in keratoconus; K1 F = flat keratometry (diopters); K2 F = steep keratometry (diopters); KI = Keratoconus Index, reflecting cone-like curvature changes; Pachy Min = minimum corneal thickness; SP-A1 = stiffness parameter at first applanation, calculated as the ratio of adjusted intraocular pressure (cIOP) to apical displacement at first applanation, reflecting corneal stiffness; SSI = Stress–Strain Index, representing the overall corneal stress–strain behavior independent of a specific stress level; TBI = Tomography and Biomechanical Index, derived from a random forest model combining Scheimpflug-based corneal tomography and biomechanical parameters.

Values are presented as mean ± standard deviation unless otherwise indicated.

*P* values from overall group comparisons and pairwise comparisons (KC vs. normal, FFKC vs. normal, KC vs. FFKC) are shown.

### Diagnostic Performance

**Figure 1:**
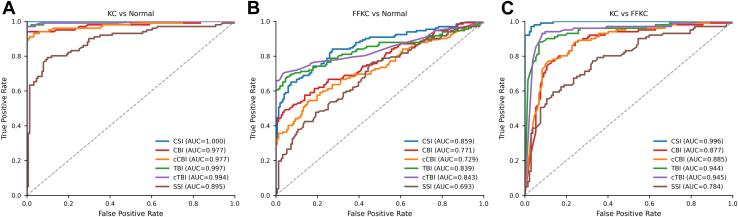
Receiver operating characteristic curves for CSI and conventional indices in pairwise diagnostic tasks. Receiver operating characteristic curves comparing the diagnostic performance of the CSI with conventional biomechanical and tomographic indices for **(A)** KC versus normal eyes, **(B)** FFKC versus normal eyes, and **(C)** KC versus FFKC. Continuous Severity Index consistently demonstrated superior or comparable performance across all tasks. AUC = area under the curve; CBI = Corvis Biomechanical Index; cCBI = Corvis Biomechanical Index for Chinese populations; cTBI = Tomography and Biomechanical Index for Chinese populations; CSI = Continuous Severity Index; FFKC = forme fruste keratoconus; KC = keratoconus; SSI = Stress–Strain Index; TBI = Tomography and Biomechanical Index.

Receiver operating characteristic analysis demonstrated that CSI consistently achieved superior discriminative performance across all pairwise diagnostic tasks (Fig 1, Table 2).

**Table 2:** Diagnostic Performance of CSI and Conventional Indices for Pairwise Classification Tasks

Parameters	AUC (95% CI)	Youden Index	Cutoff	Sensitivity	Specificity
KC vs. normal					
CBI (KC)	0.977 (0.954–0.995)	0.941	0.764	0.941	1.000
cCBI (KC)	0.977 (0.953, 0.994)	0.898	0.711	0.911	0.987
TBI (KC)	0.997 (0.991, 1.000)	0.970	0.747	0.970	1.000
cTBI (KC)	0.994 (0.983, 1.000)	0.970	0.710	0.970	1.000
CSI (KC)	1.000 (1.000, 1.000)	1.000	0.580	1.000	1.000
SSI (KC)	0.895 (0.849, 0.936)	0.693	0.604	0.792	0.901
FFKC vs. normal					
CBI (FFKC)	0.771 (0.713, 0.827)	0.446	0.719	0.492	0.954
cCBI (FFKC)	0.729 (0.668, 0.788)	0.386	0.342	0.545	0.841
TBI (FFKC)	0.839 (0.786, 0.886)	0.629	0.652	0.682	0.947
cTBI (FFKC)	0.843 (0.789, 0.891)	0.665	0.150	0.705	0.960
CSI (FFKC)	0.859 (0.814, 0.900)	0.579	0.303	0.652	0.927
SSI (FFKC)	0.693 (0.630, 0.752)	0.302	0.533	0.765	0.536
KC vs. FFKC					
CBI (FFKC)	0.877 (0.829, 0.922)	0.643	0.967	0.772	0.871
cCBI (FFKC)	0.885 (0.841, 0.927)	0.659	0.979	0.772	0.886
TBI (FFKC)	0.944 (0.913, 0.972)	0.813	0.986	0.881	0.932
cTBI (FFKC)	0.945 (0.911, 0.973)	0.847	0.940	0.931	0.917
CSI (FFKC)	0.996 (0.991, 0.999)	0.940	0.740	0.970	0.970
SSI (FFKC)	0.784 (0.725, 0.841)	0.458	0.665	0.594	0.864

AUC = area under the curve; CBI = Corvis Biomechanical Index; cCBI = Corvis Biomechanical Index for Chinese populations; CI = confidence interval; CSI = Continuous Severity Index; cTBI = Tomography and Biomechanical Index for Chinese populations; FFKC = forme fruste keratoconus; KC = keratoconus; SSI = Stress–Strain Index; TBI = Tomography and Biomechanical Index.

Receiver operating characteristic (ROC) analysis of CSI and conventional biomechanical and tomographic indices for differentiating KC, FFKC, and normal eyes. Area under the curve values with 95% CIs, Youden index, optimal cutoff values, sensitivity, and specificity are reported for each pairwise comparison.

For KC versus normal eyes, CSI achieved perfect discrimination (AUC = 1.000; 95% confidence interval [CI], 1.000–1.000), with optimal sensitivity and specificity of 100% at a cutoff value of 0.580. This performance exceeded that of all conventional indices, including CBI, cCBI, TBI, cTBI, and SSI (all *P* < 0.001).

For FFKC vs. normal eyes, CSI achieved an AUC of 0.859 (95% CI, 0.814–0.900), outperforming CBI, cCBI, and SSI, and demonstrating comparable performance to TBI (AUC = 0.839) and cTBI (AUC = 0.843). At the optimal cutoff of 0.303, CSI achieved a balanced sensitivity of 65.2% and specificity of 92.7%, highlighting its advantage in early ectasia detection.^15^

For KC versus FFKC, CSI again showed near-perfect discrimination with an AUC of 0.996 (95% CI, 0.991–0.999), achieving both sensitivity and specificity of 97.0% at a cutoff of 0.740. This performance exceeded that of biomechanical indices and remained superior to or comparable with tomographic–biomechanical composite indices.

### Distribution of CSI and Conventional Indices across Disease Stages

**Figure 2:**
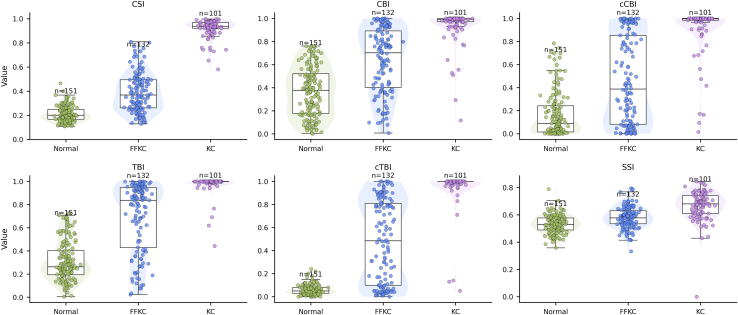
Distribution of CSI and conventional indices across KC disease stages. Distribution of the CSI and conventional biomechanical and tomographic indices across normal, FFKC, and clinical KC eyes. Continuous Severity Index demonstrates a near-monotonic increase with disease severity and minimal overlap between groups, whereas conventional indices, particularly CBI and cCBI, show substantial overlap, limiting their staging capability. CBI = Corvis Biomechanical Index; cCBI = Corvis Biomechanical Index for Chinese populations; CSI = Continuous Severity Index; cTBI = Tomography and Biomechanical Index for Chinese populations; FFKC = forme fruste keratoconus; KC = keratoconus; SSI = Stress–Strain Index; TBI = Tomography and Biomechanical Index.

As illustrated in Figure 2, CSI demonstrated a near-monotonic, stepwise increase across disease stages from normal eyes to FFKC and further to clinical KC, with minimal intergroup overlap. This clear separation highlights the ability of CSI to represent KC severity along a continuous spectrum rather than as discrete diagnostic categories.

In contrast, conventional biomechanical indices such as CBI and cCBI exhibited substantial overlap between groups, particularly between FFKC and KC, limiting their effectiveness for disease staging. Composite indices, including TBI and cTBI, showed improved separation compared with biomechanical indices alone; however, noticeable overlap persisted at adjacent disease stages.

Overall, CSI provided the most distinct stratification across normal, subclinical, and clinical KC, supporting its role as a continuous severity index capable of capturing both early ectatic changes and progressive disease advancement.

### Performance of CSI in Clinically Relevant Screening Scenarios

To evaluate the clinical utility of CSI in refractive surgery screening and disease staging, we assessed its performance in 2 clinically relevant composite classification tasks: detection of any ectatic change (KC + FFKC vs. normal) and identification of advanced disease (KC vs. FFKC + normal).

**Figure 3:**
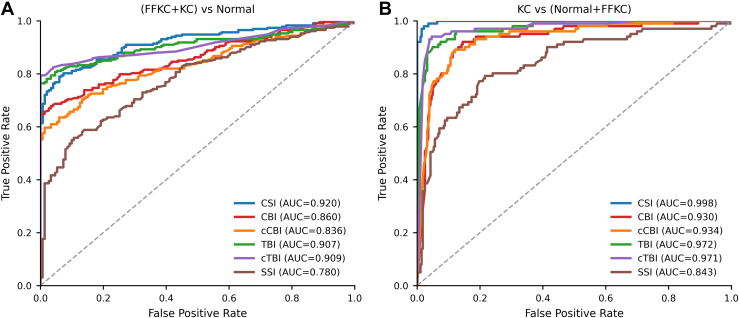
Receiver operating characteristic curves of CSI and conventional indices in composite screening tasks. Receiver operating characteristic curves comparing the performance of the CSI with conventional biomechanical and tomographic indices for **(A)** detection of any ectatic change (KC + FFKC vs. normal eyes) and **(B)** identification of advanced keratoconus (KC vs. FFKC + normal eyes). Continuous Severity Index demonstrates superior or near-perfect discriminative performance in both clinically relevant screening scenarios. AUC = area under the curve; CBI = Corvis Biomechanical Index; cCBI = Corvis Biomechanical Index for Chinese populations; CSI = Continuous Severity Index; cTBI = Tomography and Biomechanical Index for Chinese populations; FFKC = forme fruste keratoconus; KC = keratoconus; SSI = Stress–Strain Index; TBI = Tomography and Biomechanical Index.

For KC + FFKC versus normal eyes, CSI achieved an AUC of 0.920 (95% CI, 0.894–0.945), outperforming biomechanical indices including CBI, cCBI, and SSI, and demonstrating performance comparable to tomographic–biomechanical composite indices such as TBI (AUC = 0.907) and cTBI (AUC = 0.909). At the optimal cutoff of 0.311, CSI achieved a sensitivity of 79.0% and specificity of 94.0%, supporting its suitability for sensitive yet specific ectasia screening (Fig 3, Table 3).

**Table 3:** Diagnostic Performance of CSI and Conventional Indices in Composite Screening Tasks

Parameters	AUC (95% CI)	Youden Index	Cutoff	Sensitivity	Specificity
KC + FFKC vs. normal					
CBI (KC)	0.860 (0.822–0.895)	0.645	0.740	0.678	0.967
cCBI (KC)	0.836 (0.797–0.873)	0.583	0.709	0.597	0.987
TBI (KC)	0.907 (0.874–0.936)	0.764	0.729	0.764	1.000
cTBI (KC)	0.909 (0.876–0.937)	0.794	0.250	0.794	1.000
CSI (KC)	0.920 (0.894–0.945)	0.730	0.311	0.790	0.940
SSI (KC)	0.780 (0.732–0.827)	0.452	0.600	0.558	0.894
KC vs. FFKC + normal					
CBI (FFKC)	0.930 (0.897–0.959)	0.776	0.824	0.921	0.855
cCBI (FFKC)	0.934 (0.904–0.960)	0.771	0.844	0.891	0.880
TBI (FFKC)	0.972 (0.954–0.986)	0.852	0.979	0.901	0.951
cTBI (FFKC)	0.971 (0.950–0.988)	0.895	0.920	0.941	0.954
CSI (FFKC)	0.998 (0.996–1.000)	0.959	0.730	0.980	0.979
SSI (FFKC)	0.843 (0.791–0.891)	0.577	0.604	0.792	0.784

AUC = area under the curve; CBI = Corvis Biomechanical Index; cCBI = Corvis Biomechanical Index for Chinese populations; CI = confidence interval; CSI = Continuous Severity Index; cTBI = Tomography and Biomechanical Index for Chinese populations; FFKC = forme fruste keratoconus; KC = keratoconus; SSI = Stress–Strain Index; TBI = Tomography and Biomechanical Index.

Receiver operating characteristic (ROC) analysis of the Continuous Severity Index (CSI) and conventional biomechanical and tomographic indices for clinically relevant composite classification tasks: detection of any ectatic change (KC + FFKC vs. normal eyes) and identification of advanced disease (KC vs. FFKC + normal eyes). Area under the curve (AUC) values with 95% CIs, Youden index, optimal cutoff values, sensitivity, and specificity are reported.

For KC versus FFKC + normal eyes, CSI demonstrated near-perfect discrimination, with an AUC of 0.998 (95% CI, 0.996–1.000). At a cutoff value of 0.730, CSI achieved a sensitivity of 98.0% and specificity of 97.9%, outperforming all conventional indices and enabling robust identification of advanced KC requiring clinical intervention (Fig 3, Table 3).

Together, these results indicate that CSI maintains high diagnostic accuracy across both early screening and advanced disease exclusion scenarios, supporting its use as a unified severity-based index in clinical practice.

### KAN-Based Feature Attribution and Stage-Dependent Patterns

**Figure 4:**
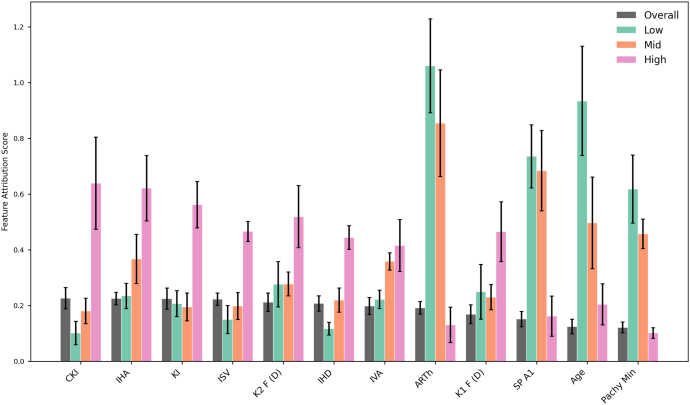
Kolmogorov–Arnold Network-based feature attribution across CSI severity stages. Global feature importance is shown for the entire dataset, along with stage-specific attributions for low (CSI < 0.33), mid (0.33–0.67), and high (CSI > 0.67) severity groups. Morphological and asymmetry-related features progressively increase with CSI, whereas biomechanical and thickness-related parameters decrease, illustrating a stage-dependent transition in dominant disease drivers. ARTh = Ambrósio relational thickness; CKI = Central Keratoconus Index; CSI = Continuous Severity Index; IHA = Index of Horizontal Asymmetry; IHD = Index of Height Decentration; ISV = Index of Surface Variance; IVA = Index of Vertical Asymmetry; KI = Keratoconus Index; SP-A1 = stiffness parameter at first applanation.

Feature attribution analysis based on the KAN-derived CSI revealed a stable global importance hierarchy across the entire dataset (Fig 4). The most influential features were CKI, IHA, KI, and ISV, followed by K2 F, IHD, IVA, and ARTh, whereas SP-A1, age, and minimum pachymetry consistently ranked lowest.

When stratified by CSI severity levels (low: <0.33; mid: 0.33–0.67; high: >0.67), a clear stage-dependent trend emerged. Several tomographic and asymmetry-related indices, including CKI, IHA, ISV, K2 F, IHD, IVA, and K1 F, demonstrated progressive increases with rising CSI values. In contrast, biomechanical and thickness-related parameters—ARTh, SP-A1, age, and minimum pachymetry—showed a marked decrease as CSI increased.

Notably, KI exhibited minimal variation between low-CSI and mid-CSI stages but increased sharply in the high CSI group, suggesting that this parameter becomes particularly informative in advanced disease states rather than early susceptibility.

## Discussion

### Principal Findings

This study introduces a Kolmogorov–Arnold Network–derived CSI for KC assessment and demonstrates its superior diagnostic performance across the full disease spectrum in a Chinese population. In particular, CSI showed robust ability to detect FFKC, a clinically challenging stage that often eludes conventional diagnostic criteria. Unlike existing indices that rely on discrete classification thresholds,^16^ CSI provides a unified, continuous representation of disease severity, which more closely reflects the gradual biological progression of KC.

### Biomechanical Alterations Precede Morphological Changes

Consistent with previous reports,^17^ our findings support the concept that corneal biomechanical weakening precedes overt tomographic abnormalities in early ectatic disease. Biomechanical parameters such as Ambrósio relational thickness (ARTh) and the stiffness parameter at first applanation (SP-A1) showed substantial deviation from normal values in FFKC eyes, despite relatively preserved geometric structure. These observations reinforce the hypothesis that biomechanical instability represents an early permissive factor in KC development and highlight the importance of incorporating biomechanical information into early screening strategies.

### Value of Continuous Severity Modeling

The progression from normal cornea to subclinical and then clinical KC occurs along a continuum that is inadequately captured by binary or ternary classification systems. The CSI addresses this limitation by embedding categorical diagnostic labels into a continuous latent severity space, enabling finer-grained risk stratification. This property is particularly valuable^18^ in refractive surgery screening, where borderline cases frequently challenge clinical decision-making and carry significant consequences for patient safety.

### Methodological Advantages of KAN

KANs^19^ offer a distinct methodological advantage over conventional machine learning models by parameterizing feature interactions through learnable spline functions. This design allows KANs to model smooth, nonlinear relationships, while retaining feature-level interpretability. As a result, CSI not only achieves strong diagnostic performance but also enables direct interrogation of how individual parameters contribute to disease severity across stages.

### Stage-Dependent Feature Contributions and Pathophysiologic Insight

The observed stage-dependent feature attribution patterns provide mechanistic insight into KC progression as captured by CSI. Indices related to corneal asymmetry and curvature geometry, including CKI, IHA, and ISV, progressively increased in importance with rising CSI values, reflecting advancing structural deformation. In contrast, biomechanical and thickness-related parameters such as ARTh and SP-A1 contributed most prominently at lower CSI levels and declined in relative influence as disease severity increased.

This divergence suggests a biologically coherent transition in disease drivers: biomechanical weakening facilitates early susceptibility, whereas geometric and asymmetry-based changes dominate later disease expression and staging. Notably, the Keratoconus Index (KI) remained relatively stable in early stages but increased sharply at high CSI values, supporting its role as a marker of manifest KC^20^ rather than early subclinical change. Together, these findings reinforce the validity of CSI as a continuous severity index grounded in KC pathophysiology rather than a purely data-driven classifier.

### Clinical Implications

The CSI has the potential to function as a unified diagnostic and staging tool within routine clinical workflows. In screening contexts that prioritize sensitivity, CSI effectively identifies early ectatic changes, including FFKC. Conversely, in confirmatory settings requiring high specificity, CSI reliably distinguishes advanced KC. This dual capability may help reduce false-negative screening outcomes, while avoiding unnecessary exclusion of suitable refractive surgery candidates.

### Limitations and Future Directions

This study is limited by its single-center retrospective design and lack of external validation. Although FFKC was defined using fellow-eye criteria, label uncertainty remains inherent in subclinical KC^20^ research. Future studies should incorporate multicenter, multi-ethnic cohorts and longitudinal progression endpoints to validate CSI as a prognostic biomarker. Integration with longitudinal progression modeling may further extend CSI toward risk prediction and treatment guidance.

## Conclusions

We present a novel, interpretable, and continuous KC severity index derived using Kolmogorov–Arnold Networks.^19^ By transcending traditional categorical diagnostics, CSI enables improved early detection, refined disease staging, and biologically meaningful interpretation across the full spectrum of KC. This framework holds significant promise for refractive surgery screening and KC management and may serve as a generalizable paradigm for modeling other progressive ocular diseases.
